# Clinical efficacy of video double-lumen tube for one-lung ventilation in thoracic surgery: a meta-analysis of randomized controlled trials

**DOI:** 10.3389/fmed.2026.1819031

**Published:** 2026-05-21

**Authors:** Li Jiang, Xiaoqian Wu, Yue Yuan, Yi Li, Ge Yang, Dong Zhang

**Affiliations:** Department of Anesthesiology, Hebei General Hospital, Shijiazhuang, Hebei, China

**Keywords:** double-lumen tube, meta-analysis, one-lung ventilation, thoracic surgery, video double-lumen tube

## Abstract

**Background:**

Double-lumen tube (DLT) is generally regarded as the gold standard for one-lung ventilation during thoracic surgery. Compared with DLT, video double-lumen tube (VDLT) has an integrated camera, allowing continuous visualization of its position in the trachea. However, the clinical efficacy of VDLT for one-lung ventilation in thoracic surgery is unclear. This meta-analysis aims to evaluate the clinical efficacy of VDLT for one-lung ventilation in thoracic surgery.

**Methods:**

The PubMed, EMBASE, Web of Science, the Cochrane Library, CNKI, WanFang, and CBM databases were searched for relevant studies from inception to July 2025. This meta-analysis used RevMan and Stata software to implement statistical analysis. The primary outcome was the intubation time. Sensitivity analysis and Egger’s test were performed to evaluate the stability of the results and the publication bias.

**Results:**

Eighteen studies involving 1,332 patients were included. For the primary outcome intubation time, extreme and unexplained between-study heterogeneity was observed (I^2^ = 99%). A random-effects model was applied, and the pooled result suggested a trend toward shorter intubation time in the VDLT group (MD = −102.68 [95% CI: −123.71 to −81.66], *p* < 0.00001). Meta-analysis also revealed that the VDLT group exerted a higher first-attempt success rate (OR = 8.04, 95% CI [2.17 to 29.74], *p* = 0.002), lower mean arterial pressure (MD = −16.85 [95% CI: −21.67 to −12.02], *p* < 0.00001) and heart rate (MD = −11.32 [95% CI: −19.80 to −2.85], *p* = 0.009) after intubation, a shorter repositioning time after dislodgement (MD = −50.91 [95% CI: −67.30 to −34.52], *p* < 0.00001), and a lower incidence of sore throat (OR = 0.62, 95% CI [0.39 to 0.98], *p* = 0.04). No significant differences regarding the incidence of dislodgement during positioning and surgery, quality of lung collapse, or the incidence of hoarseness after surgery were noted between the 2 group.

**Conclusion:**

Compared with the DLT, VDLT may provide modest clinical advantages of a shorter intubation time, higher first-attempt success rate, lower mean arterial pressure and heart rate after intubation, shorter repositioning time after dislodgement, and lower incidence of sore throat. However, the primary outcome of intubation time is limited by extreme between-study heterogeneity that could not be explained. Standardized multicenter international randomized trials are warranted to confirm the clinical efficacy of VDLT.

**Systematic review registration:**

https://www.crd.york.ac.uk/PROSPERO/view/CRD420251138139, Identifier: CRD420251138139.

## 1 Introduction

In order to expose the surgical area while maintaining normal ventilation in the non-operative lung, one of the essential features of thoracic surgery is the requirement for mechanical ventilation separation between the two lungs (1). The most popular method of one-lung ventilation is the use of double-lumen tube (DLT), which has the benefits of a relatively secure tube location and a significant impact on lung collapse (2). However, several studies revealed that even with skilled caregivers, a substantial prevalence of DLT malposition necessitated recurrent repositioning (3). Additionally, thoracic surgical patients frequently have limited tolerance for apnea and poor lung function. Therefore, for anesthetic to be administered safely during one lung ventilation, the DLT must be positioned correctly.

The use of video double-lumen tube (VDLT) for lung isolation is constantly increasing as visualization technology advances (4). In order to provide continuous surveillance and monitoring of endotracheal tube installation, the VDLT is designed similarly to the standard DLT, which has an implanted camera and light source between the tracheal and bronchial cuffs (5). It was reported that the utilization of VDLT was related to favorable cost-effectiveness (3). Continuous visualization with a VDLT, as opposed to a DLT, could greatly lessen the requirement for a fiberoptic bronchoscope (FOB) and offer continuous monitoring during the surgical operation, enabling early detection of tube displacement (6). Furthermore, there have been many reports on the use of VDLT in one-lung ventilation in recent years. Nonetheless, the validity and consistency of VDLT remain unclear (7, 8).

Therefore, we systematically searched the related articles and conducted this meta-analysis with the aim of exploring the clinical efficacy of VDLT in one-lung ventilation in thoracic surgery and providing a reference for clinical practice.

## 2 Methods

Ethical approval was not required for this study, as it is a meta-analysis.

### 2.1 Study registration

To ensure data quality, this meta-analysis was conducted in accordance with the Preferred Reporting Items for Systematic Reviews and Meta-Analysis (PRISMA) guidelines (9). This study was registered in the International Prospective Register of Systematic Reviews (PROSPERO; identifier: CRD 420251138139).

### 2.2 Literature research

Two researchers carried out a comprehensive, language-neutral literature search in the electronic databases of PubMed, EMBASE, Web of Science, the Cochrane Library, CNKI, WanFang, and CBM up to July 2025 without restrictions on language. Supplementary Table 1 displays the specifics of the search approach. To guarantee a thorough search, the researchers also manually examined the most recent reviews’ references to determine if they satisfied the inclusion requirements. Any disagreements were settled by dialogue and agreement.

### 2.3 Study selection

After scanning the aforementioned databases, two authors independently examined the titles, abstracts, and full texts of the remaining publications in order to choose the final studies. Where there was disagreement, a conclusion was reached after debate.

The PICOS framework was used to include pertinent research: (1) P (patients), adult patients receiving thoracic surgery with one-lung ventilation; (2) I (interventions), VDLT for intubation; (3) C (control), DLT for intubation; (4) O (outcome), intubation time, first-attempt intubation success rate, hemodynamic parameters including mean arterial pressure and heart rate, repositioning time, tube dislodgement, lung collapse quality, and postoperative complications such as sore throat and hoarseness; (5) S (study) randomized controlled trials (RCTs).

The following were among the study’s exclusion criteria: (1) repeated publications; (2) case reports, reviews, protocols and non-RCTs.

### 2.4 Data extraction

Two seasoned researchers separately extracted the following parameters: first author, year of publication, country of study, sample size, participant age, ASA physical status, outcome indicators, and definition of intubation time. The primary outcome was determined to be the intubation time. First-attempt success rate, mean arterial pressure (MAP) and heart rate (HR) before and after intubation, the incidence of dislodgement during positioning and surgery, repositioning time following dislodgement, lung collapse quality, as well as postoperative sore throat and hoarseness incidence were the secondary outcomes. The median (interquartile) data were transformed into mean and standard deviation using the formula Luo et al. (10) presented.

### 2.5 Quality assessments

Risk of bias was assessed inde- pendently by two authors (YL and GY) using the Cochrane risk-of-bias tool for randomised trials (ROB-2) (11). This tool evaluates five domains: (1) the randomization process, (2) deviations from intended interventions, (3) missing outcome data, (4) outcome measurement, and (5) selection of the reported result. We assessed all domains for each outcome across different studies, and judged each domain according to these risk-of-bias ratings: “low risk,” “some concerns,” or “high risk.”

### 2.6 Certainty of the evidence assessment

We constructed a summary of findings table using GRADEpro GDT software, and by following Cochrane guidance. The summary of findings table included the relative and absolute effects and.

the certainty of the evidence for all outcomes (see Supplementary Table 2). Two review authors (YL, GY), working independently and in duplicate, applied GRADE criteria to evaluate the certainty of the evidence for each outcome. We resolved conflicts by discussion. If we were unable to reach a consensus, we consulted another review author (DZ).

### 2.7 Statistical analysis

The program used for the statistical analysis was RevMan v. 5.4.1 and Stata 15.0. For dichotomous outcomes, we computed the odds ratio (OR) and 95% confidence interval (CI); for continuous data, we computed the mean difference (MD) and 95% CI. Higgins I^2^ and the associated *p*-value were used to assess for heterogeneity. When I^2^ > 50% or *p* < 0.1, a random-effects model was utilized; otherwise, a fixed-effects model was applied. Subgroup analyses and meta-regression were conducted to explore potential sources of heterogeneity. The funnel plot and Egger’s test were also used to assess the potential for publication bias. If there was publication bias, the trim-to-fill method was used to adjust it. Sensitivity analysis was conducted using the leave-one-out technique. A *p*-value of less than 0.05 was considered a statistically difference.

## 3 Results

### 3.1 Study selection

**Figure 1:**
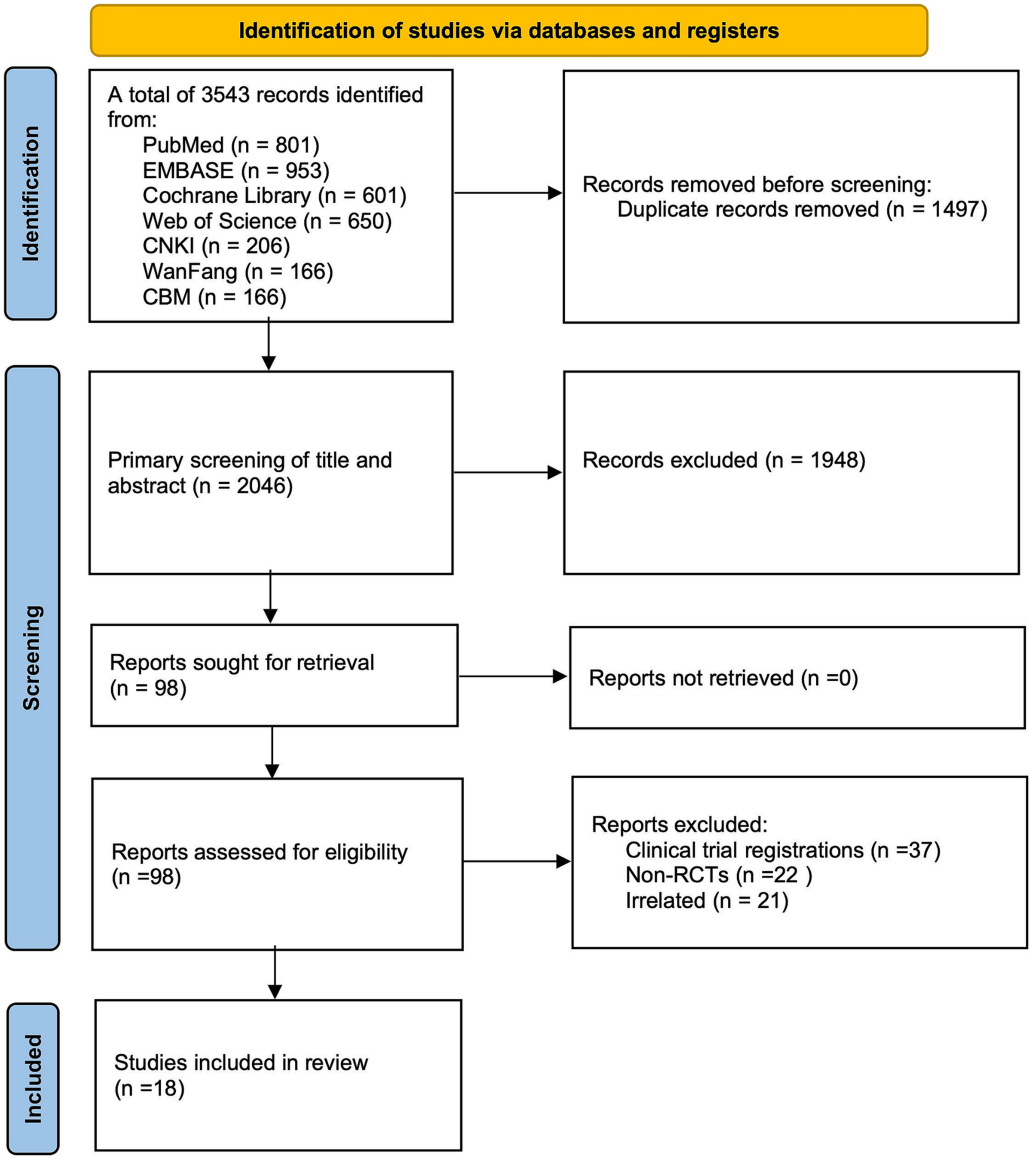
The PRISMA flow diagram of study selection for the current meta-analysis.

3,543 studies were first pulled from the databases by two authors, and 1,497 were removed since they were duplicates. 1948 papers that did not meet inclusion criteria were eliminated after two additional investigators examined the abstracts and titles. After a full-text review of the remaining 98 publications, those that satisfied the exclusion criteria were excluded. Ultimately, 18 studies in total were selected for this meta-analysis (7, 8, 12–27) (Figure 1).

### 3.2 Description of included studies

Table 1 provided a summary of the enrolled trials’ parameters. All publications were published between May 2015 and November 2024, and their sample sizes ranged from 48 to 182. The research was conducted across multiple countries, including China, Israel, Poland, Switzerland, and the United States of America.

**Table 1:** Summary of the included studies’ characteristics.

Author and year	Country	Age (sample size)		ASA physical status	Outcome indicators	Definition of intubation time
		DLT	VDLT			
Chen et al. 2024 (12)	China	54.27 ± 7.14 (40)	51.90 ± 5.12 (40)	I~III	①③④⑥⑦⑨⑩	The duration from the exposure of the glottis with a video laryngoscope to the moment when accurate positioning enables single-lung ventilation.
Fei et al. 2019 (13)	China	48.19 ± 8.16 (52)	47.84 ± 7.94 (52)	Not mentioned	①②⑨	Not mentioned
Fu et al. 2021 (14)	China	42 ± 5.6		Not mentioned	①②⑦	The duration from the laryngoscope passes through the patient’s lips until the anesthesia machine display shows three complete exhalation-ventilation cycles of carbon dioxide.
		(25)	(25)			
Heir et al. 2017 (15)	USA	67.97 ± 4.3		I~IV	①⑤⑥⑨	The duration from the placement of laryngoscope until visual confirmation on external monitor or end-tidal carbon dioxide.
		(42)	(38)			
Huang et al. 2020 (16)	China	57.5 ± 10.8 (30)	52.2 ± 10.7 (30)	I~III	①②⑨⑩	The duration from the insertion of the DLT through the glottis until the attending physician reports to the chief resident that the DLT has successfully located the target bronchus.
Jing et al. 2018 (17)	China	52.36 ± 5.88 (30)	56.51 ± 6.33 (30)	I~III	①②③④⑧⑨⑩	The time required for DLT to pass through the glottis and reach the catheter for accurate positioning.
Levy-Faber et al. 2015 (7)	Israel	67 [61–75] (36)	68 [61–74] (35)	I~IV	①⑨⑩	The duration from introducing the laryngoscope blade until the DLT position was visually verified.
Li et al. 2018 (18)	China	60 ± 12 (30)	65 ± 9 (30)	II~III	①③④	The duration from the insertion of the tracheal tube into the main airway to the successful positioning and entry into the bronchus.
Li et al. 2018 (19)	China	Not mentioned		I~III	①⑨⑩	The duration from the insertion of the direct laryngoscope into the throat to the detection of the end-tidal carbon dioxide waveform.
		(40)	(40)			
Liu et al. 2023 (20)	China	54.3 ± 13.2 (29)	55.1 ± 13.7 (28)	I~III	①⑥⑧⑨⑩	The duration from the removal of the tube core through the glottis of the blue cuff to the confirmation of a satisfactory position
Lv et al. 2018 (21)	China	52 ± 10 (30)	54 ± 12 (30)	I~II	①②⑥⑦	The duration from the removal of the tube core through the glottis of the blue cuff to the confirmation of a satisfactory position
Onifade et al. 2020 (22)	USA	55 [43–61] (25)	57 [42–65] (25)	II~IV	①⑤⑥⑧	The time from the start of laryngoscopy to the time that the bronchial lumen of the DLT was successfully visualized within the left mainstem bronchus.
Palaczynski et al. 2022 (23)	Poland	60 ± 17 (39)	63 ± 14 (32)	Not mentioned	①⑤⑥⑨⑩	The time from the beginning of direct laryngoscopy to the proper placement of DLT in the respective groups.
Schuepbach et al. 2015 (24)	Switzerland	63 ± 10 (20)	57 ± 17 (20)	I~III	①⑤⑥⑧⑨⑩	The time from insertion of the laryngoscope to confirmation ofplacement by auscultation.
Shi et al. 2021 (25)	China	58.0 ± 13.9 (24)	58.7 ± 9.7 (24)	II~III	①③④⑥⑦⑨⑩	Not mentioned
Shui et al. 2024 (8)	China	59.5 ± 11.0 (90)	58 ± 12.5 (92)	I~III	①②⑧⑨⑩	The duration between the laryngoscope lifting of the epiglottis and the first time at which no further manipulation of the (V)DLT was needed.
Zhao et al. 2017 (26)	China	Not mentioned		I~III	①②③④⑤⑥⑧⑨⑩	The time required for DLT to pass through the larynx and reach the guide tube and achieve satisfactory positioning.
		(40)	(40)			
Zhou et al. 2018 (27)	China	50.2 ± 12.1 (50)	51.2 ± 11.2 (50)	I~III	①⑥⑦⑨⑩	The time required for the double-lumen tube to pass through the glottis until the catheter positioning is satisfactory.

USA, the United States of America; DLT, double-lumen tube; VDLT, video double-lumen tube; ASA, American Society of Anesthesiologists; ① Intubation time; ② First-attempt success rate; ③ Mean arterial pressure before and after intubation; ④ Heart rate before and after intubation; ⑤ Dislodgement during positioning; ⑥ Dislodgement during surgery; ⑦ Repositioning time following dislodgement; ⑧ Lung collapse quality; ⑨ Sore throat; ⑩ Hoarseness incidence.

### 3.3 Primary outcome

#### 3.3.1 The impact of VDLT on intubation time

**Figure 2:**
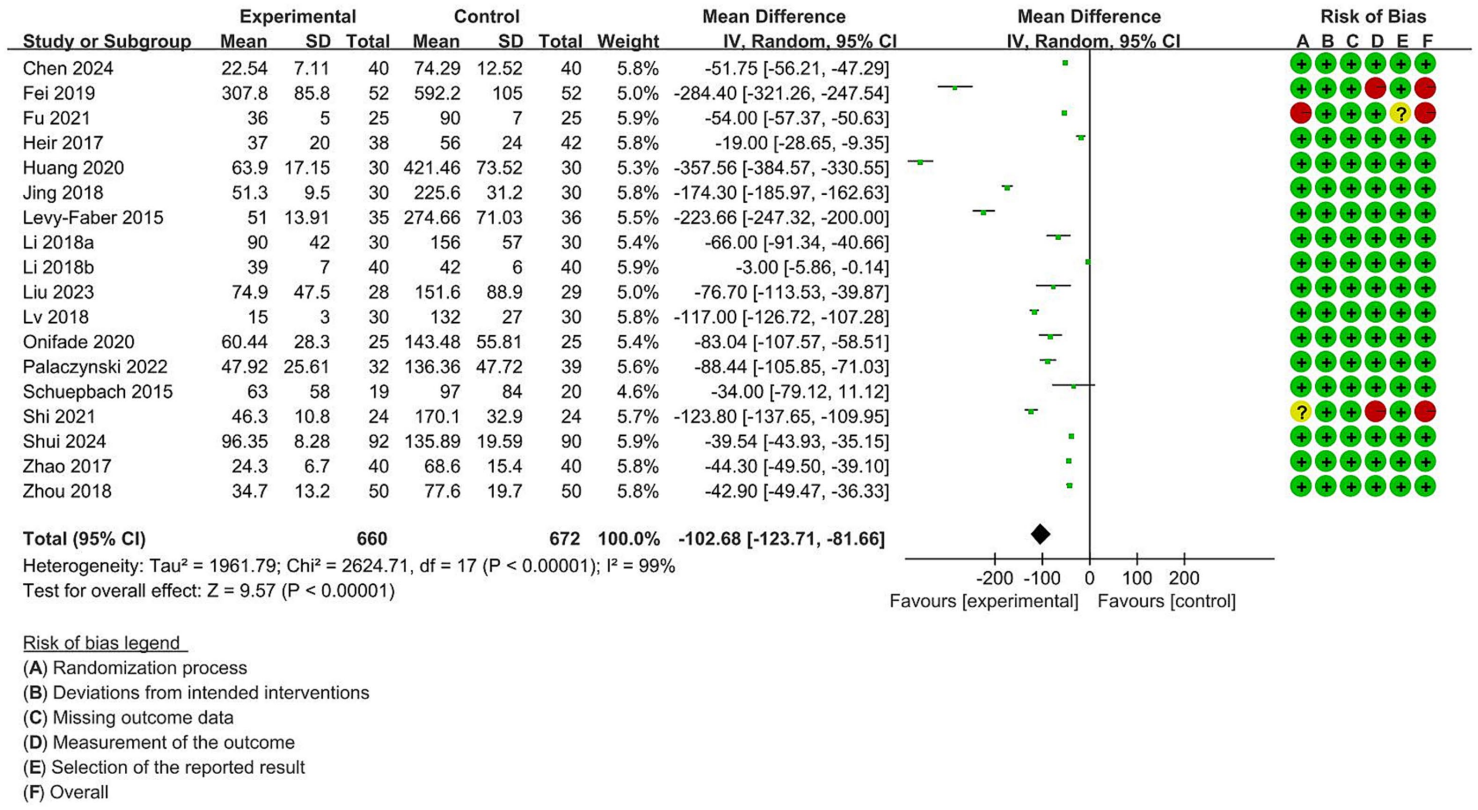
Forest plot for the impact of video double-lumen tube (VDLT) on intubation time. Cochrane ROB-2 results are also presented. SD, Standard deviation; IV, inverse variance; CI, confidence interval; df, degree of freedom.

The intubation time analysis covered 18 trials with 1,332 individuals in total. High study heterogeneity (I^2^ = 99%) led to the use of a random-effects model to minimize statistical error. The results showed that compared with DLT, VDLT could shorten the intubation time (MD = -102.68 [95% CI: −123.71 to −81.66], *p* < 0.00001) (Figure 2). Notably, subgroup analyses and meta-regression did not explain the high heterogeneity. The funnel plot was asymmetric (Supplementary Figure 1), and Egger’s regression test was significant (*p* < 0.01), indicating that significant publication bias was found in our study. Next, we further adjusted the publication bias using a trim-to-fill analysis, and the results revealed that 3 articles were missing (Supplementary Figure 2). After adding the potential missing 3 studies, the combined results did not change significantly (MD = −120.69 [95% CI: −168.66 to −72.72], *p* < 0.00001), suggesting that the outcome of our meta-analysis was reliable. Additionally, sensitivity analysis demonstrated that removing a single study had no significant effect on the intubation time (Supplementary Figure 3).

### 3.4 Secondary outcomes

#### 3.4.1 First-attempt success rate

**Figure 3:**
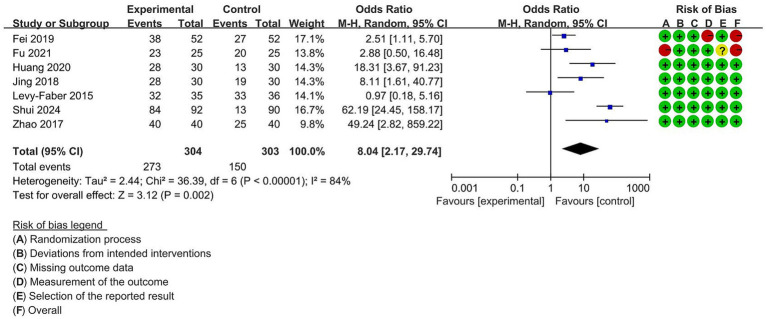
Forest plot for the impact of video double-lumen tube (VDLT) on the first-attempt success rate. Cochrane ROB-2 results are also presented. M-H, Mantel–Haenszel; CI, confidence interval; df, degree of freedom.

The first-attempt success rate was reported in seven studies with 607 participants. Because there was high heterogeneity (I^2^ = 84%) among the research, we decided to apply random-effects model to lower the statistical error. As presented in Figure 3, the VDLT group had a higher first-attempt success rate in comparison with the DLT group (OR = 8.04, 95% CI [2.17 to 29.74], *p* = 0.002).

#### 3.4.2 MAP and HR before and after intubation

**Figure 4:**
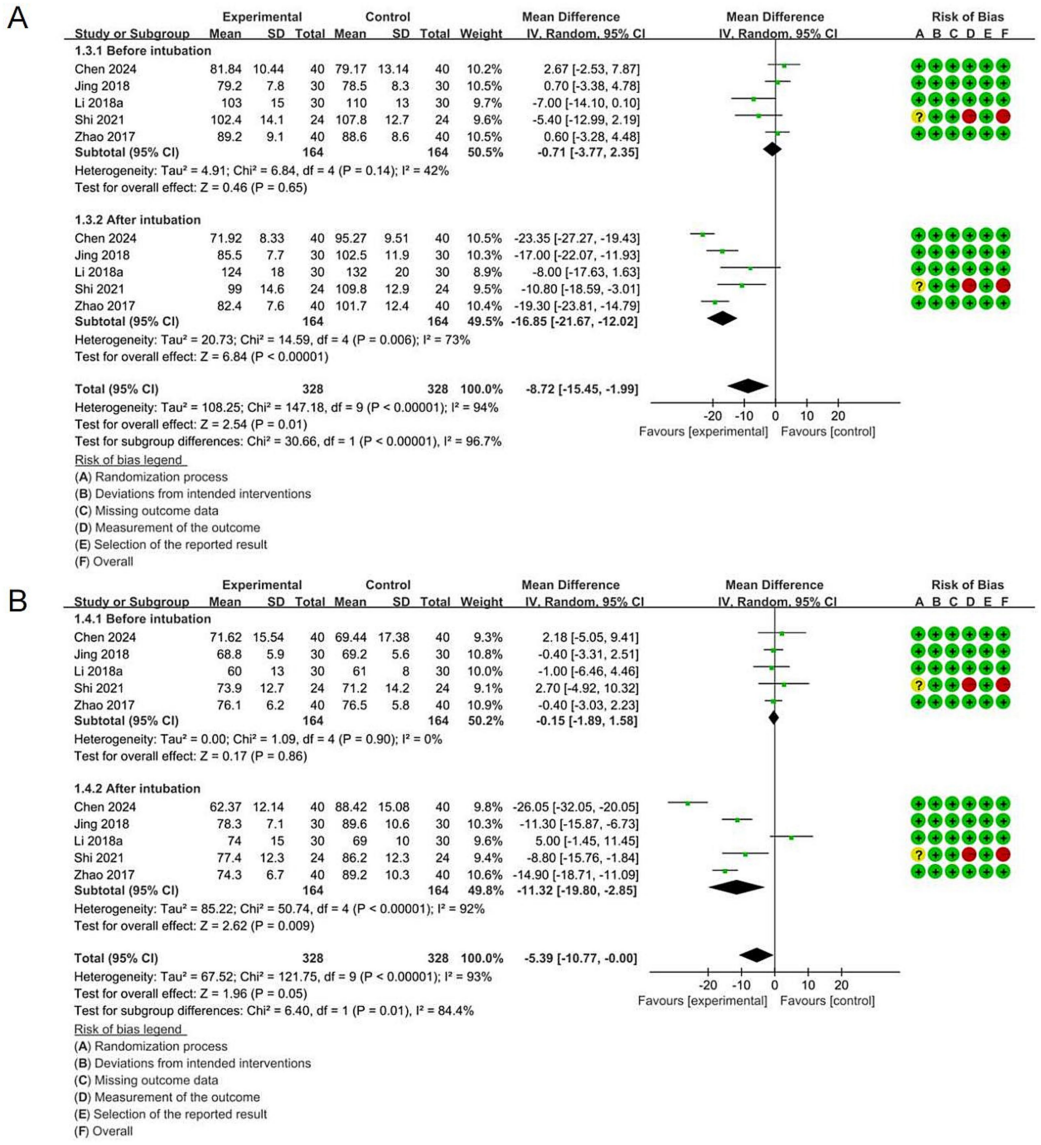
Forest plot for the impact of video double-lumen tube (VDLT) on hemodynamic before and after intubation: **(A)** Mean arterial pressure (MAP); **(B)** Heart rate (HR). Cochrane ROB-2 results are also presented. SD, standard deviation; IV, inverse variance; CI, confidence interval; df, degree of freedom.

Five studies (656 participants) were available for this analysis. Before intubation, there was no significant difference between VDLT and DLT in terms of MAP (MD = −0.71 [95% CI: −3.77 to 2.35], *p* = 0.65) and HR (MD = −0.15 [95% CI: −1.89 to 1.58], *p* = 0.86). However, compared with DLT, the VDLT group showed lower MAP (MD = -16.85 [95% CI: −21.67 to −12.02], *p* < 0.00001) and HR (MD = −11.32 [95% CI: −19.80 to −2.85], *p* = 0.009) after intubation. A random-effects model was applied because of the high heterogeneity (Figure 4).

#### 3.4.3 The incidence of dislodgement during positioning and surgery

**Figure 5:**
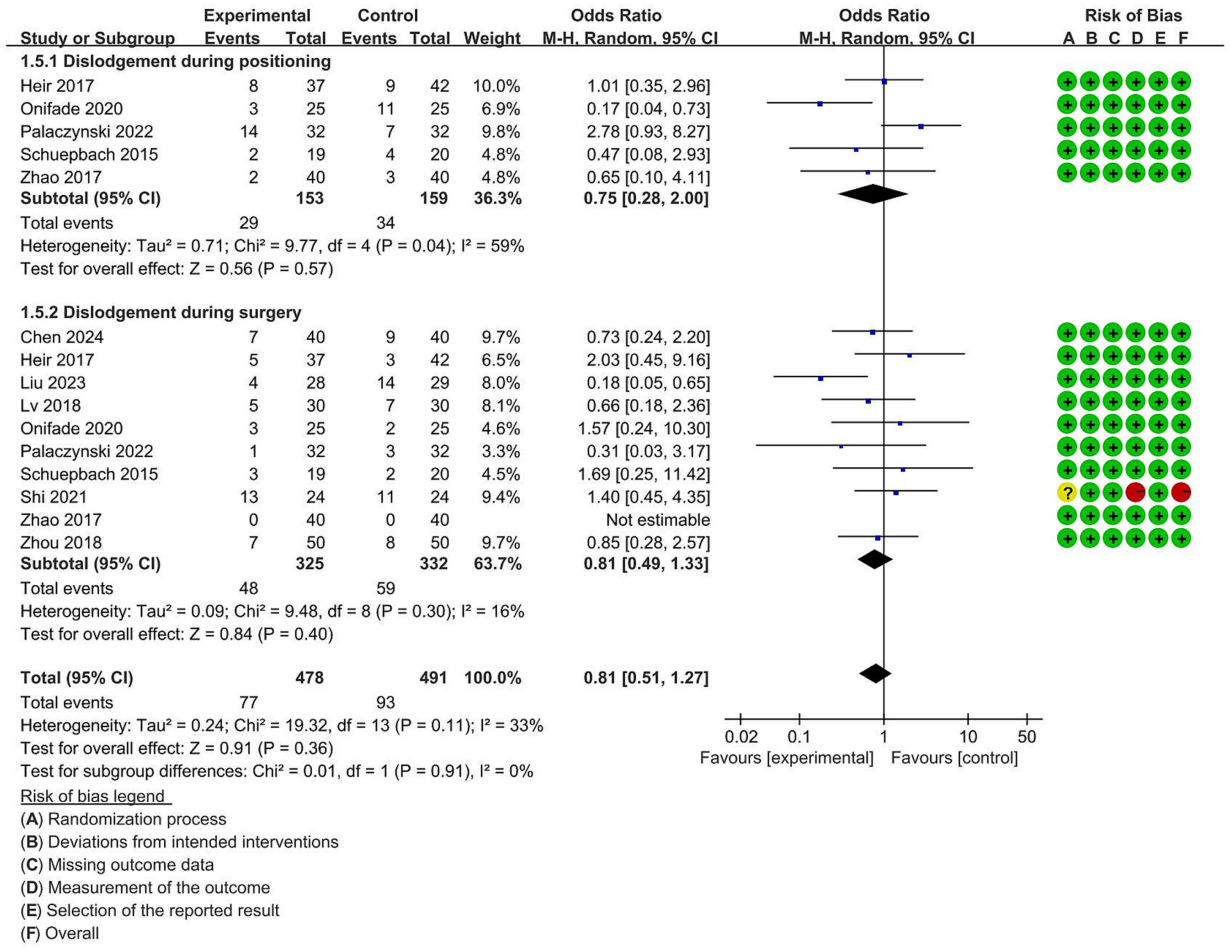
Forest plot for the impact of video double-lumen tube (VDLT) on the incidence of dislodgement during positioning and surgery. Cochrane ROB-2 results are also presented. M-H, Mantel–Haenszel; CI, confidence interval; df, degree of freedom.

Five studies reported the incidence of dislodgement during positioning, and 10 studies investigated the dislodgement during surgery. The pooled analysis showed no significant difference in the incidence of dislodgement during positioning (OR = 0.75, 95% CI [0.28 to 2.00], *p* = 0.57) and surgery (OR = 0.81, 95% CI [0.49 to 1.33], *p* = 0.40) between VDLT and DLT group. A random-effects model was chosen to reduce statistical error (Figure 5).

#### 3.4.4 Repositioning time following dislodgement

**Figure 6:**
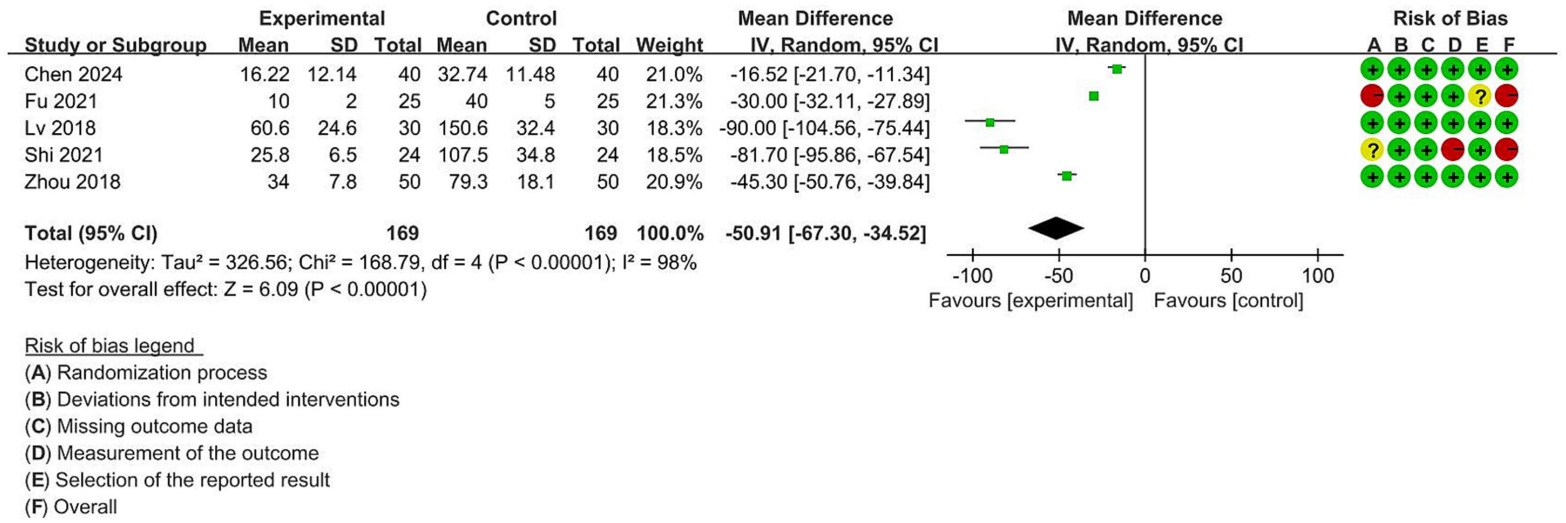
Forest plot for the impact of video double-lumen tube (VDLT) on the repositioning time following dislodgement. Cochrane ROB-2 results are also presented. SD, standard deviation; IV, inverse variance; CI, confidence interval; df, degree of freedom.

The repositioning time was reported in 5 trials with 338 participants. Since high heterogeneity was observed among these studies (I^2^ = 98%), the random-effects model was utilized. As illustrated in Figure 6, compared with the DLT group, the VDLT group required a shorter repositioning time after dislodgement (MD = −50.91 [95% CI: −67.30 to −34.52], *p* < 0.00001).

#### 3.4.5 Quality of lung collapse

**Figure 7:**
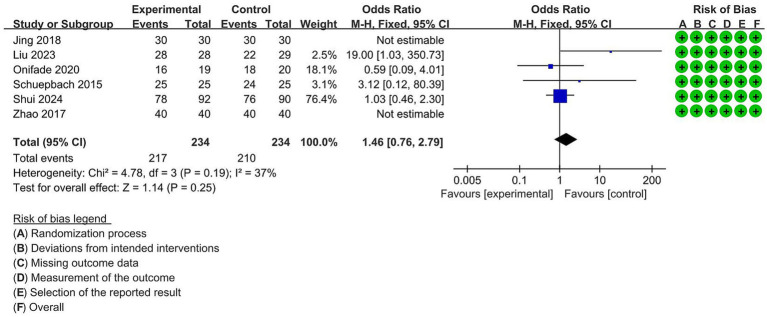
Forest plot for the impact of video double-lumen tube (VDLT) on the lung collapse quality. Cochrane ROB-2 results are also presented. M-H, Mantel–Haenszel; CI, confidence interval; df, degree of freedom.

Six studies with 468 participants reported the quality of lung collapse. The researchers of these studies rated the extent of the lung collapse as follows: excellent complete collapse with perfect surgical exposure; fair (total collapse but some residual air in the lung); poor (no or partial collapse with possible interference during the surgical procedure). In this meta-analysis, we calculated the incidence of excellent lung collapse, and the result indicated that there was no statistically difference between the VDLT group and the DLT group (OR = 1.46, 95% CI [0.76 to 2.79], *p* = 0.25). Due to the low heterogeneity, we adopted a fixed-effect model to report the result (I^2^ = 37%) (Figure 7).

#### 3.4.6 The incidence of sore throat and hoarseness after surgery

**Figure 8:**
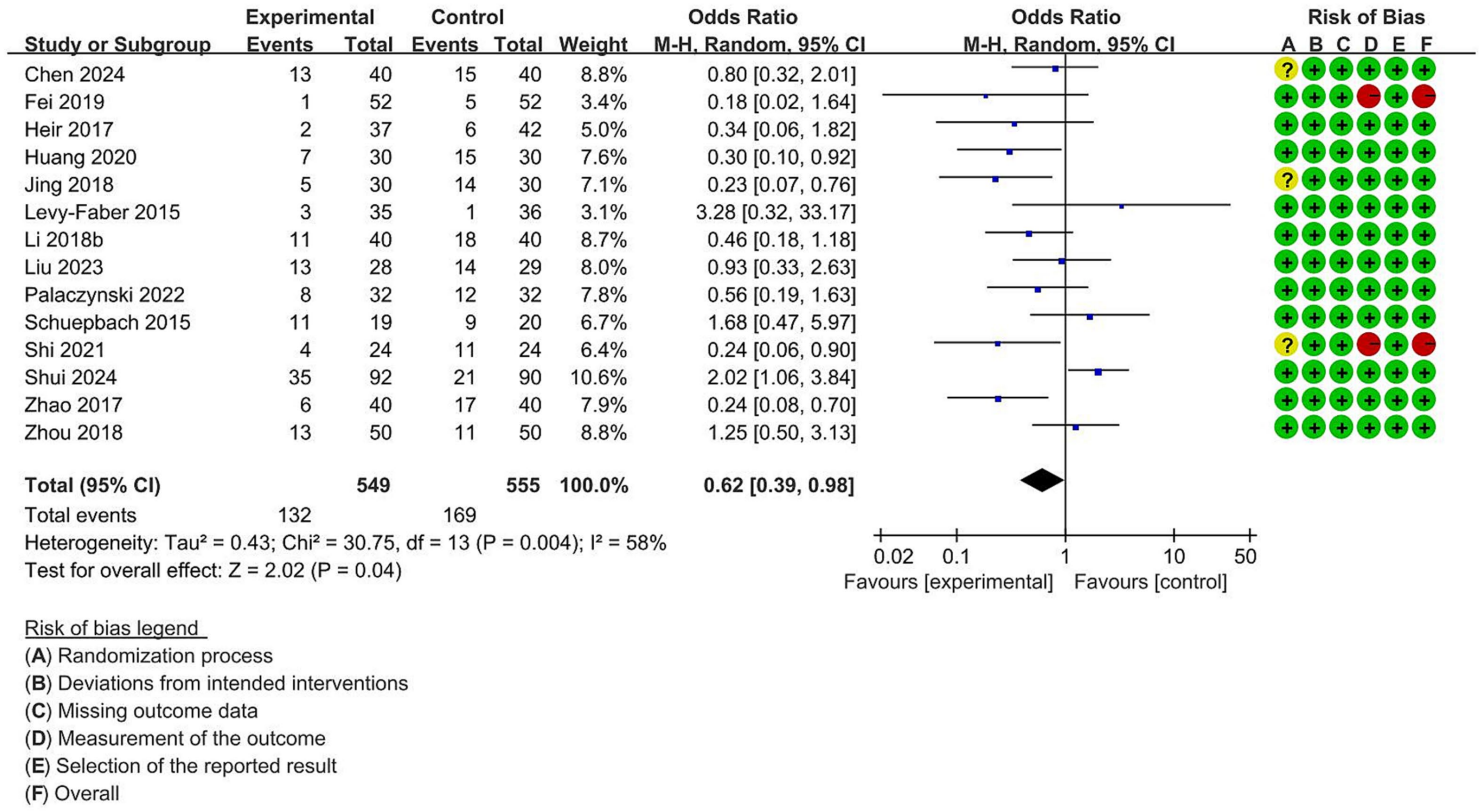
Forest plot for the impact of video double-lumen tube (VDLT) on the postoperative sore throat incidence. Cochrane ROB-2 results are also presented. M-H, Mantel–Haenszel; CI, confidence interval; df, degree of freedom.

**Figure 9:**
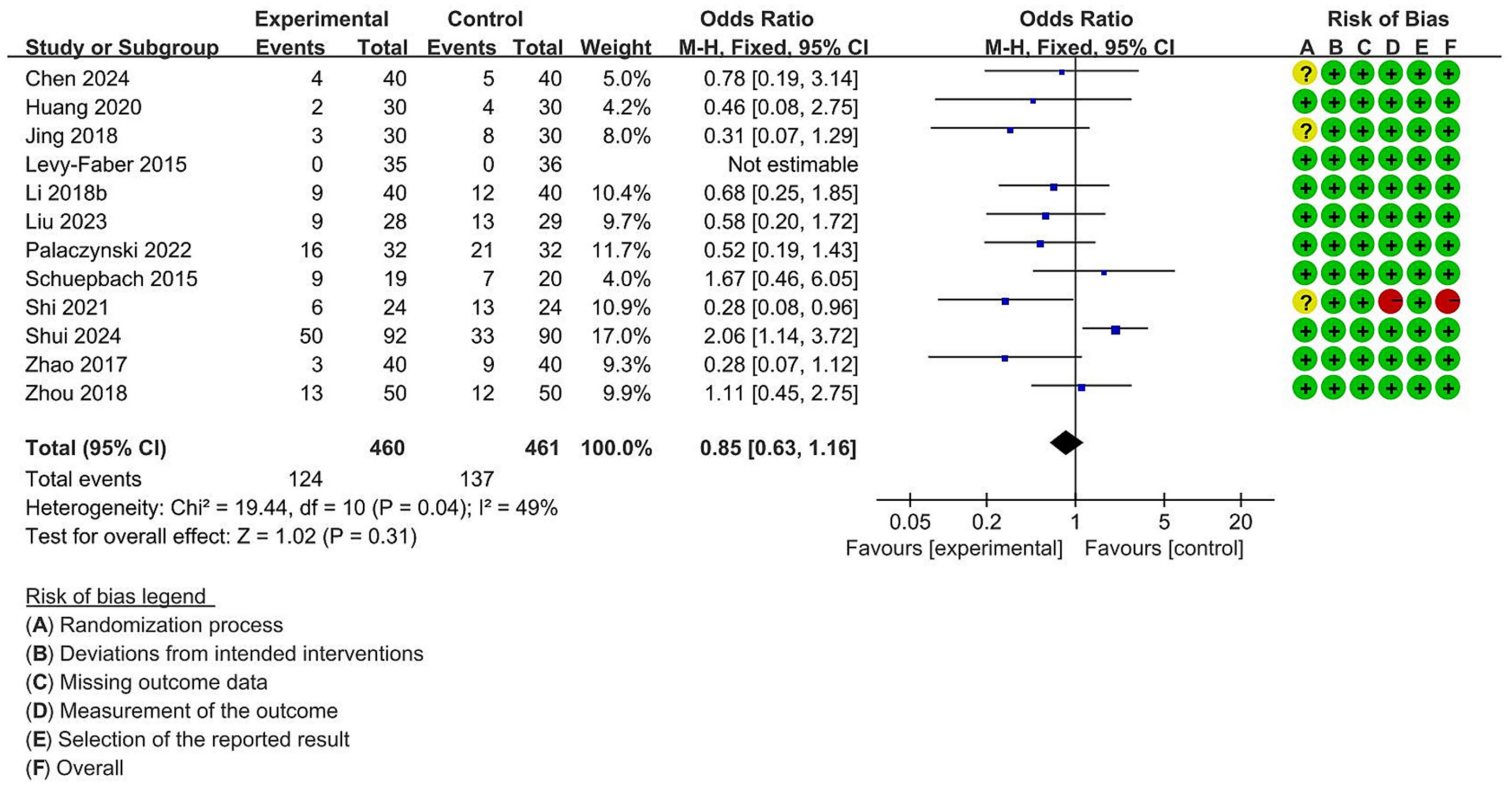
Forest plot for the impact of video double-lumen tube (VDLT) on the postoperative hoarseness incidence. Cochrane ROB-2 results are also presented. M-H, Mantel–Haenszel; CI, confidence interval; df, degree of freedom.

A meta-analysis of 14 studies with 1,104 participants showed a significantly lower incidence of sore throat after surgery (OR = 0.62, 95% CI [0.39 to 0.98], *p* = 0.04) in the VDLT group (Figure 8). A random-effects model was performed because of significant heterogeneities among trials (I^2^ = 58%). Twelve studies reported the incidence of hoarseness after surgery. A fixed-effects model was chosen due to no meaningful heterogeneity among the trials (I^2^ = 49%). As shown in Figure 9, no significant statistical difference was identified between the two groups (OR = 0.85, 95% CI [0.63 to 1.16], *p* = 0.31).

## 4 Discussion

This systematic review and meta-analysis involving 1,332 patients demonstrated that compared with DLT, the use of VDLT during thoracic surgery with one-lung ventilation resulted in shorter intubation time, higher first-attempt success rate, lower MAP and HR after intubation, shorter repositioning time after dislodgement, and lower incidence of sore throat.

Patients undergoing thoracic surgery often suffer from severe respiratory insufficiency. Prolonged intubation time might lead to hypoxia and carbon dioxide retention. Thus, it has become a significant concern for anesthesiologists to select an optimal lung isolation technique that enables rapid and effective establishment of single-lung ventilation. Our findings indicated that the VDLT group achieved significantly shorter intubation time and higher first-attempt intubation success rate compared to the DLT group. This disparity might stem from the blind nature of DLT, which compromises placement accuracy and often necessitates secondary catheter guidance via FOB. Conversely, VDLT eliminates the need for FOB verification due to its direct distal visualization. Notably, this result exhibited extreme and unexplained heterogeneity (I^2^ = 99%), which could not be resolved by subgroup analyses, meta-regression, or sensitivity analysis. This substantial heterogeneity most likely reflects inconsistent definitions of intubation time, varying operator experience, anesthetic protocols, and institutional practices across studies. Despite the high heterogeneity, random-effects modeling was used to provide a conservative summary estimate. However, the pooled result should be viewed only as a descriptive trend and interpreted with great caution. Overall, VDLT may reduce the duration of no-ventilation time compared to conventional method, enhancing safety during anesthesia procedures. However, due to the high heterogeneity, this finding should be carefully interpreted.

It was pointed out that the reduction of endotracheal intubation duration could mitigate the stimulation to hemodynamics, lessen the inflammatory stress response, and stabilize hemodynamics (28). Due to the abundant sensory nerves in the pharynx, the placement of a DLT could cause airway irritation, leading to excessive sympathetic nervous system excitation and the over-release of catecholamines, resulting in elevated blood pressure and increased HR (29). The results of this study indicated that before intubation, MAP and HR were comparable between the VDLT group and the DLT group, while patients in the VDLT group experienced stable hemodynamic fluctuations after intubation. Previous studies demonstrated that excessively high blood pressure and HR significantly increased the incidence of perioperative cardiovascular and cerebrovascular events (30). Therefore, employing VDLT to maintain circulatory stability during the induction phase of anesthesia held significant clinical importance.

Thoracic surgery requires patient repositioning, and surgical manipulation might cause traction or compression of the bronchial tree, leading to dislodgement of DLT. The occurrence of dislodgement might cause rapid desaturation or re-expansion of the operative lung, affecting surgical procedures, and even might allow secretions from the diseased lung to enter the healthy lung, posing risks to surgery and anesthesia (14). Our findings showed no statistically significant difference in the number of patients experiencing dislodgement during positioning and surgery between the two groups, consistent with previous studies (23). Once tube displacement is detected intraoperatively, repositioning is required. Our result indicated that the VDLT group required less time for repositioning than the DLT group. This might be attributed to the VDLT’s simpler procedure, which does not require the use of a FOB or video laryngoscope, thereby enhancing anesthetic efficiency.

Additionally, OLV of thoracic surgery requires complete collapse of the non-ventilated lung in order to provide an adequate surgical field. Inadequate collapse of lung could make surgical manipulation extremely difficult (31). In this study, there was no statistically significant difference between the two groups in the incidence of excellent lung collapse, indicating that the safety and efficacy of VDLT were comparable to DLT.

Postoperative sore throat and hoarseness are common airway complications following DLT intubation (32). The results of this study indicate that the use of VDLT reduced the incidence of sore throat in patients. As previously reported, the application of VDLT enabled faster and more precise placement into the main bronchus, avoiding repeated manipulation that irritated the posterior pharyngeal wall, thereby lowering the incidence of pharyngeal injury (25). Furthermore, the present study found no significant difference between the two groups regarding the incidence of postoperative hoarseness. Research demonstrated that the incidence and severity of postoperative hoarseness could be influenced by multiple confounding factors, including operator intubation technique and tube diameter (33). In the future, more methods need to be explored to alleviate patients’ adverse experiences.

Of particular importance for visual airway devices such as VDLT is the potential risk of thermal injury to the bronchial mucosa. A large-sample clinical trial was prematurely terminated due to severe thermal damage caused by overheating of the impeded camera tip during prolonged airway instrumentation (34). This adverse event highlights a critical safety concern that direct visualization devices may generate excessive heat when the tip is obstructed or in prolonged contact with airway mucosa, potentially leading to iatrogenic bronchial burns. Therefore, while VDLT offers clear advantages in intubation efficiency and hemodynamic stability, clinicians should remain vigilant regarding device positioning, avoid prolonged contact of the camera tip with bronchial tissue, and ensure adequate heat dissipation during use to prevent thermal mucosal injury.

It was also important to note the limitations in this meta-analysis. First, extreme and unexplained heterogeneity (I^2^ = 99%) was noted for the primary outcome of intubation time. Although random-effects models were used to improve generalizability, the underlying clinical and methodological differences could not be fully eliminated. Second, nearly all studies were unable to blind providers to the randomization of the DLT or VDLT, which could result in response bias. Third, the sample size of the studies included in this meta-analysis was relatively small. Therefore, our findings ought to be regarded with caution and should be confirmed in high-quality RCTs with a large sample size.

## 5 Conclusion

In summary, the utilization of the VDLT in thoracic surgery with one-lung ventilation enables rapid intubation positioning, delivers more stable hemodynamics, provides continuous and effective monitoring with rapid repositioning, and has a lower incidence of sore throat. However, the primary outcome of intubation time is limited by extreme between-study heterogeneity that could not be explained. Standardized multicenter international randomized trials are warranted to confirm the clinical efficacy of VDLT.

## Data Availability

The original contributions presented in the study are included in the article/Supplementary material, further inquiries can be directed to the corresponding author.
