# Supplementary material for: Clinical efficacy of video double-lumen tube for one-lung ventilation in thoracic surgery: a meta-analysis of randomized controlled trials
Source: Front Med (Lausanne). 2026 May 21;13:1819031. doi: 10.3389/fmed.2026.1819031 (PMC13233436; doi:10.3389/fmed.2026.1819031)
Supplement: Supplementary file 1 [file Data_sheet_1.docx]

**Supplementary Table 1. Each database retrieval strategy**

**PUBMED**

| **Search** | **Query** | **Results** |
| --- | --- | --- |
| # 1 | one-lung ventilation[MeSH Terms] OR one lung ventilation[Title/Abstract] OR ventilation, one-lung[Title/Abstract] OR single-lung ventilation[Title/Abstract] OR single lung ventilation[Title/Abstract] OR single-lung ventilations[Title/Abstract] OR ventilation, single-lung[Title/Abstract] OR ventilations, single-lung[Title/Abstract] OR lung separation techniques[Title/Abstract] OR lung separation technique[Title/Abstract] OR separation technique, lung[Title/Abstract] OR separation techniques, lung[Title/Abstract] OR technique, lung separation[Title/Abstract] OR techniques, lung separation[Title/Abstract] OR thoracic surgery[MeSH Terms] OR thoracoscopic surgery[Title/Abstract] OR video-assisted thoracoscopy[Title/Abstract] OR thoracotomy[Title/Abstract] OR lung surgery[Title/Abstract] OR lobectomy[Title/Abstract] OR lung resection[Title/Abstract] OR pulmonary resection[Title/Abstract] | 433983 |
| # 2 | video double-lumen tube[Title/Abstract] OR vivaSight double-lumen tube[Title/Abstract] OR double-Lumen tube[Title/Abstract] OR VDLT[Title/Abstract] OR DLT[Title/Abstract] | 4561 |
| # 3 | #1 AND #2 | 801 |

**Supplementary Table 2. GRADE certainty assessment (video double-lumen tube vs. double-lumen tube) using GradePro software.**


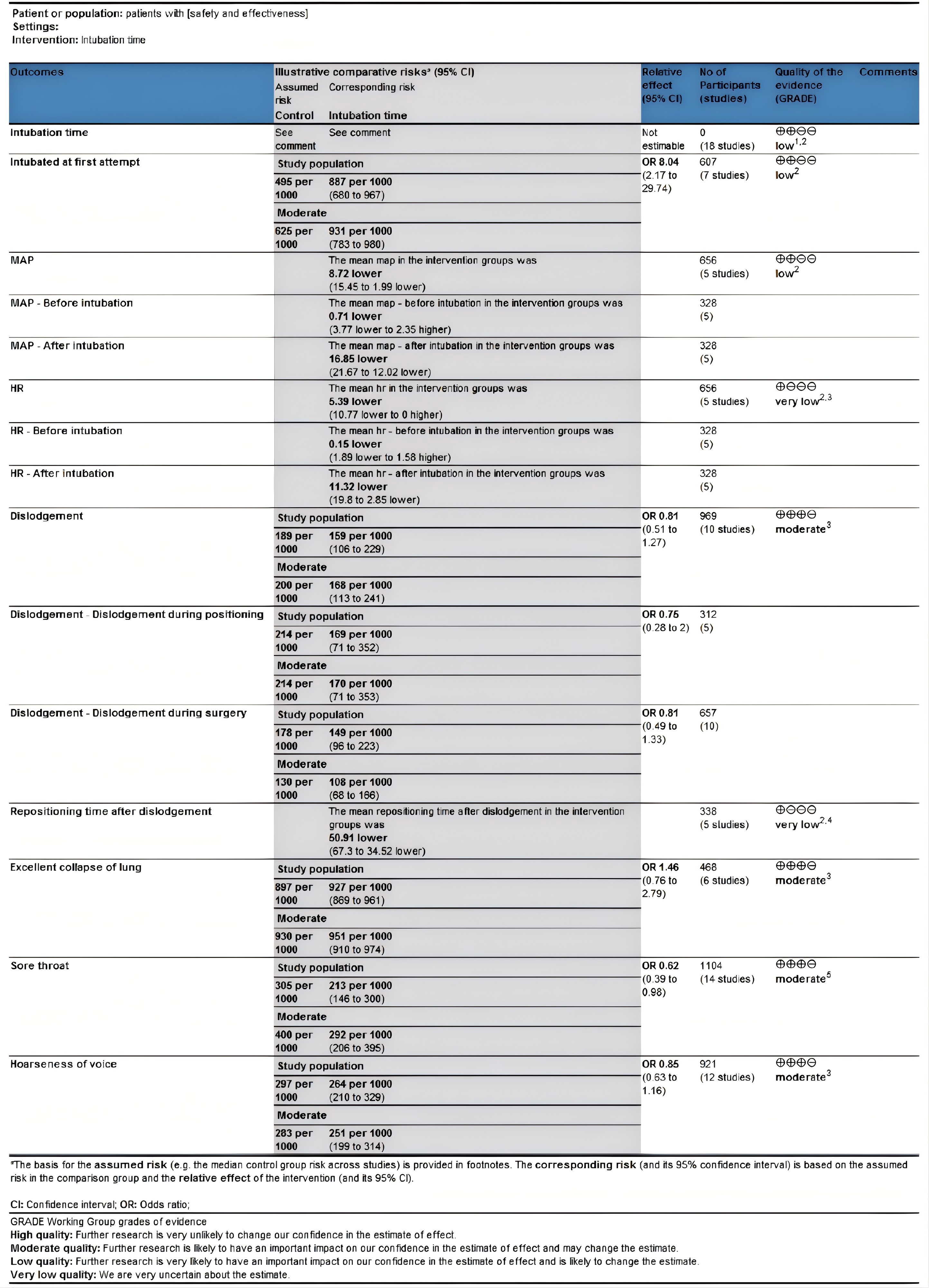

Figure 1 Funnel plot for the impact of video double-lumen tube (VDLT) on intubation time, compared with double-lumen tube (DLT). SMD, standard mean difference

Figure 2 The sensitivity analysis of the impact of video double-lumen tube (VDLT) on intubation time, compared with double-lumen tube (DLT).
